# A New Spectrophotometric Method for Determination of Selenium in Cosmetic and Pharmaceutical Preparations after Preconcentration with Cloud Point Extraction

**DOI:** 10.1155/2011/729651

**Published:** 2011-04-05

**Authors:** Mohammad Hosein Soruraddin, Rouhollah Heydari, Morteza Puladvand, Mir Mehdi Zahedi

**Affiliations:** ^1^Department of Analytical Chemistry, Faculty of Chemistry, Tabriz University, Tabriz 51664-14766, Iran; ^2^Department of Chemistry, Faculty of Science, Islamic Azad University, Khorramabad Branch, Khorramabad, Iran; ^3^Department of Analytical Chemistry, Faculty of Chemistry, Razi University, Kermanshah, Iran

## Abstract

A simple, rapid, and sensitive spectrophotometric method for the determination of trace amounts of selenium (IV) was described. In this method, all selenium spices reduced to selenium (IV) using 6 M HCl. Cloud point extraction was applied as a preconcentration method for spectrophotometric determination of selenium (IV) in aqueous solution. The proposed method is based on the complexation of Selenium (IV) with dithizone at pH < 1 in micellar medium (Triton X-100). After complexation with dithizone, the analyte was quantitatively extracted to the surfactant-rich phase by centrifugation and diluted to 5 mL with methanol. Since the absorption maxima of the complex (424 nm) and dithizone (434 nm) overlap, hence, the corrected absorbance, Acorr, was used to overcome the problem. With regard to the preconcentration, the tested parameters were the pH of the extraction, the concentration of the surfactant, the concentration of dithizone, and equilibration temperature and time. The detection limit is 4.4 ng mL^−1^; the relative standard deviation for six replicate measurements is 2.18% for 50 ng mL^−1^ of selenium. The procedure was applied successfully to the determination of selenium in two kinds of pharmaceutical samples.

## 1. Introduction

Selenium (Se) has been recognized as an essential nutrient for plant, animal, and human body, but at high concentration it can become toxic. The range between the concentration in which selenium is essential and toxic is very narrow [1, 2]. This element plays an important role in elderly people as well as in the prevention of many age-associated diseases and in maintenance of normal immune function. Se is potent antioxidant involved in cellular defense against free radical reactions, and the risk of deficiency seems to increase in proportion to the age. Evidence is accumulating that most of the degenerative diseases have their origin in deleterious free radical reactions. These diseases include atherosclerosis, cancer, inflammatory joint, asthma, diabetes, senile dementia, and degenerative eye disease [3]. But high Se concentrations in human can cause loosing hair and nails and irritation of skin and eye [1]. Se is essential nutrient for human health. The required daily amount of Se is 20 mcg day^−1^ and 40–70 mcg day^−1^ for 4–6 years old and adult males, respectively. The essential role of the Se is due to its presence in the active site of some enzymes (i.e., glutathione peroxidase and iodotironine-5-deiodinase) and the catalytic effect of selenium compounds on the reaction of intermediate metabolism and inhibition of the toxic effect of heavy metals. It has been established that diets with deficit in Se are associated with some human diseases, but diets with Se contents higher than 5 mg/kg are toxic and cause important symptoms in humans and animals [4]. Selenium is present as selenocysteine (Se-Cys) in at least 30 proteins [5]. All these factors, together with the fact that the concentration levels are extremely low, call for sensitive and accurate method for the determination of this element. 

Selenium can be present in −2, 0, +4, and +6 oxidation states. These forms cannot simultaneously be evaluated by direct application of certain analytical techniques such as instrumental neutron activation analysis (INAA) [6, 7], as well as hydride generation atomic absorption spectroscopy (HG-AAS), inductively coupled plasma atomic emission spectrometry (ICP-AES), electrothermal atomic absorption spectrometry (ET-AAS), or inductively coupled plasma mass spectrometry (ICP-MS), after digestion of sample [8–10]. The most common methods used for determination of various species of selenium were atomic absorption spectrometry (hydride generation and electrothermal atomization) [11–14], molecular [15, 16] and atomic fluorescence spectrometry [17–19], high-performance liquid chromatography (HPLC) [20–22], voltammetry [23, 24], atomic emission spectrometry (AES), such as inductively coupled plasma (ICP) [2] and inductively coupled plasma-mass spectrometry (ICP-MS) [20], and spectrophotometry methods [25–28]. In spite of some of the above methods, spectrophotometric methods are popular because of their simplicity and are based on piazselenol complex formation between the reagents and selenium. 

An interesting alternative to traditional liquid-liquid extraction is the micelle-mediated extraction, firstly developed by Watanabe and Tanaka [29]. The cloud point is the temperature above which aqueous solutions of nonionic and zwitterionic surfactant become turbid. Micelles of such well-known nonionic surfactant as Triton X-100 or X-114 have a nonpolar core and extended polar layer, where both extractants and extracted complexes can be solubilized. Above the cloud point, the solution is separated into two phases, a rich phase containing a high surfactant concentration in a small volume and a poor phase with a surfactant concentration close to the critical micelle concentration (CMC). Hydrophobic species (hydrophobic organic compounds or metal ions after reaction with a suitable hydrophobic ligand) present in sample are able to interact with the micelles, thus being concentrated in the small volume of the surfactant-rich phase. This simple procedure is called cloud point extraction (CPE) [30–33].

The aim of this work was to use a simple, rapid, and sensitive spectrophotometric method (dithizone as chromogenic reagent) for determination of trace amounts of selenium (IV) (5–100 ng mL^−1^) in micellar medium (Triton X-100) in cosmetic and pharmaceutical products. To improve the detection limit of this method, cloud point extraction (CPE) has been used.

## 2. Experimental

### 2.1. Material and Chemicals

Selenium metal (purity: 99.9%) was obtained from the Riedel de Haen (Germany). Nitric acid (>99.5%), sodium hydroxide (>97%), ascorbic acid, hydrochloric acid (36.5–38%), phenol (>99.0%), methanol (>99.8%), and sulfuric acid (95.0–97.0%) were purchased from Merck (Germany). Dithizone was supplied by Searle (UK). All chemicals were used without any further purification. Deionized water from a Milli-Q system (Millipore, USA) was used for preparation of all solutions. Selenium sulphide shampoo (Iran), Centrum tablet (USA), and Selen plus capsule (Germany) were obtained from market.

### 2.2. Instrumentation

A Shimadzu double-beam UV-Vis spectrophotometer (model 1650 PC, Japan) with 1.0 cm quartz cell was used for all spectral measurements. In order to investigate the accuracy of method a PerkinElmer (model Optima 7000 DV) inductively coupled plasma optical emission spectrometer (ICP-OES) was used as reference method. Separation of surfactant phase and aqueous phase was carried out with a Hettich centrifuge (EBA 20, UK). A microwave system (Microdigest 301, Prolabo, France) with a maximum irradiation power of 200 W was used. pH measurements were made with a Metrohm pH meter (model 744, Switzerland). The effect of temperature was investigated by a GFL thermostated bath (model 1003, Germany).

### 2.3. Preparation of Solutions

The stock standard solution of selenium (IV) (1000 *μ*g mL^−1^) was prepared by dissolving 100 mg of selenium metal in hot concentrated nitric acid and diluted to 100 mL with water [25]. From this solution serial dilutions were made to obtain different concentration levels of 5, 15, 30, 40, 55, 70, 80, and 100 ng mL^−1^ of selenium (IV). The stock solution of dithizone (10^−3^ mol l^−1^) was prepared daily by dissolving 12.8 mg of the reagent in 5 mL of NaOH solution (0.1 M, containing the 50 mg of ascorbic acid for stabilizing the dithizone) and diluting to 50 mL with water. The stock solution was diluted with water to obtain the final dithizone concentration of about 7.5 *μ*mol l^−1^. The stock solution of Triton X-100 (10% (w/v)) was prepared by weighing 10 g of this reagent into 100 mL volumetric flask, dissolving in water by gentle heating, and making up to the mark with water. The final concentration of Triton X-100 (0.2%) was prepared by suitable dilution of stock solution with the diluent (solution of 0.5% (w/v) of phenol).

### 2.4. Procedure

**Figure 1:**
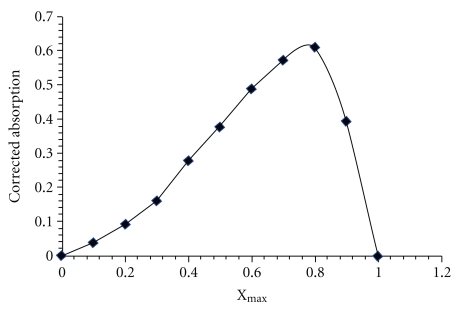
Job plot for determination of Se(IV)/dithizone ratio.

An aliquot of 50 mL of a solution containing selenium (IV) (final concentration 5–100 ng mL^−1^), Triton X-100 (0.2% (w/v)), dithizone (7.5 *μ*mol l^−1^), and HCl (0.35 M) was kept for 15 min to complete the color development. Then the mixture was placed in a water bath at 40°C for 10 min, when 0.5% (w/v) of the phenol was used for inducing the cloud point. Phase separation was accelerated by centrifuging the test tube at 3500 rpm for 15 min. The bulk aqueous phases were easily decanted by gently inverting the test tubes, and the surfactant-rich phase (complex rich) was made up to 5 mL by adding methanol. The same procedure was applied for preparation of blank solution without the selenium and dithizone. The absorbance of two methanolic solutions was measured at 424 and 592 nm, against a blank (prepared in the same way without Se and dithizone). The absorption maxima of the complex (424 nm) and dithizone (434 nm) were overlapping (Figure 1). Hence, the corrected absorbance, *A*_corr_, was used to overcome the problem [34]:

(1) $$A_{\text{corr}}=A_{424}-{\left(\frac{A_{424}}{A_{592}}\right)}_{L}\times A_{592},$$

where *A*_424_ and *A*_592_ are the absorbance of the complex measured at 424 and 592 nm, respectively, and (*A*_424_/*A*_592_)_*L*_ is the absorbance ratio of dithizone at 424 and 592 nm. The complex does not show any absorbance at 592 nm. *A*_corr_ gives the real absorbance of the complex. Under optimum conditions, a calibration graph was used for determination of selenium in cosmetic and pharmaceutical products.

### 2.5. Preparation of Real Samples

Shampoo (Selenium sulphide, Iran) sample dissolution was prepared using the procedure described by Afkhami et al. [25]. Approximately 1 g of shampoo sample was weighted into a 100 mL beaker. Concentrated sulfuric acid (1 mL) was added, and the mixture was heated to fuming for 15 min. The solution was allowed to cool, and 5 mL of 30% (w/v) hydrogen peroxide was added. The mixture boiled vigorously to eliminate excess hydrogen peroxide and allowed to cool and then the digest transferred to a 100 mL volumetric flask. The solution was diluted to the mark with water. Finally, 2 mL of this solution was taken for determination of selenium (IV) as in the recommended procedure. 

A Centrum tablet (USA) containing 0.25 mg Se (as Sodium Selenate) was placed in digestion vessel. 5 mL of concentrated nitric acid and 2 mL of hydrogen peroxide were added and the mixture subjected to microwave irradiation (25% of power, 5 min) in order to digest the sample. Then, 10 mL of hydrochloric acid (6 M) was added, and the mixture was irradiated by microwave (75% of power, 5 min) in order to reduce all Se to Se (IV) [4]. The mixture was then diluted to 100 mL with water. 1 mL of treated sample solution was dropwise passed through an Eichrom cation exchange resin (sulfonic acid groups, mesh ranges: 50–100 *μ*m, 8 cm), for removal of interferences, and selenium was determined with the recommended procedure. 

A Selen plus capsule (Germany) was dissolved in 10 mL HCl (6 M), and the mixture was irradiated by microwave (75% of power, 5 min) in order to reduce all Se to Se (IV). The mixture was then diluted to 50 mL with water, and then 1 mL of this mixture was taken for determination of selenium (IV) as in the recommended procedure.

## 3. Results and Discussion

### 3.1. Composition and Stability Constant of the Complex

From the plot obtained by Job's method of continuous variation, the Se- (IV) to- dithizone (H_2_DZ) ratio forming the complex was found to be 1 : 4 at pH < 1. The complex can, therefore, be represented as Se(HDZ)_4_ which is in agreement with the reported composition in the organic phase [35]. The conditional stability constant of the 1 : 4 (Se : H_2_DZ) complex was calculated, assuming the following equilibrium in the system:

(2) $${\text{H}}_{2}{\text{SeO}}_{3}+4\;{\text{H}}_{2}\text{DZ}⟷\text{Se}{\left(\text{HDZ}\right)}_{4}+3{\text{H}}_{2}\text{O}.$$

Using the plot obtained by Job's method, X_max _(C_HDZ_/C_Total_) was found to be 0.8, and therefore the ratio of Se : HDZ was calculated 1 : 4 (Figure 1). The stability constant was found to be 1.13 × 10^22^.

**Figure 2:**
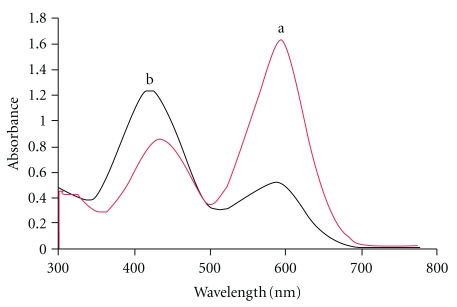
Absorption spectra of (a) dithizone (HDZ) in methanol, (b) Se (IV)-HDZ complex. Conditions: 0.2% (w/v) Triton X-100, 7.5 *μ*mol l^−1^ HDZ, 0.35 M HCl, 100 ng mL^−1^ Se (IV), equilibration time: 15 min, and equilibration temperature: 40°C.

Selenium (IV) reacts with dithizone (HDZ) in micellar medium and forms a hydrophobic complex, Se(HDZ)_4_, which is subsequently trapped in the surfactant micelles and separated from the aqueous phase.  Figure 2 shows the absorption spectra for the dithizone and selenium-dithizonate complex in surfactant-rich phase against a reagent blank. The absorption maximum of the complex was 424 nm, and the absorption maxima of the dithizone were 434 and 592 nm. The complex does not show any absorbance at 592 nm.

### 3.2. Optimization of Selenium (IV) Dithizonate Complex Formation Effect of HCl Concentration

**Figure 3:**
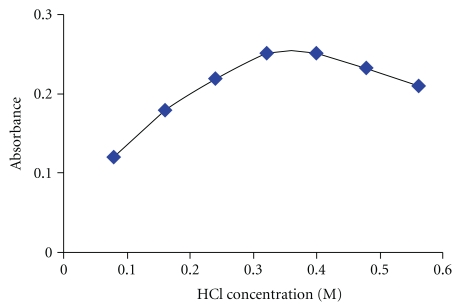
Effect of HCl concentration on the corrected absorbance of the complex. Conditions: 0.2% (w/v) Triton X-100, 7.5 *μ*mol l^−1^ HDZ, equilibration time: 15 min, and equilibration temperature: 40°C and 50 ng mL^−1^ of Se (IV).

Selenium reacts with dithizone in acidic pH [35]. Solution pH plays a unique role on metal-chelate function and subsequent extraction.  Figure 3 shows the influence of HCl concentration on the corrected absorbance of the selenium dithizonate complex at 424 nm. As can be seen, the complex extraction was increased with the increased HCl concentration and then showed a decrease. The reason for this behaviour might be because of decreasing the dithizone stability at higher pHs. Hence, an HCl concentration of 0.35 M was chosen for further studies.

### 3.3. Effect of Dithizone Concentration

**Figure 4:**
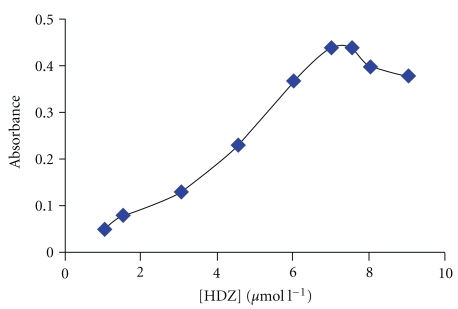
Effect of HDZ concentration on the analytical response. Conditions: 0.2% (w/v) Triton X-100, 0.35 M HCl, equilibration time: 15 min, equilibration temperature: 40°C, and 50 ng mL^−1^ Se (IV).

The influence of the dithizone (HDZ) concentration on analytical response is shown in Figure 4. This figure shows that the absorbance increased up to a known concentration of dithizone (7.5 *μ*mol l^−1^) and then reached a plateau that can be attributed to complete extraction. Therefore, the concentration of 7.5 *μ*mol l^−1^ of dithizone was selected as the optimum. 

### 3.4. Effect of Triton X-100 Concentration

**Figure 5:**
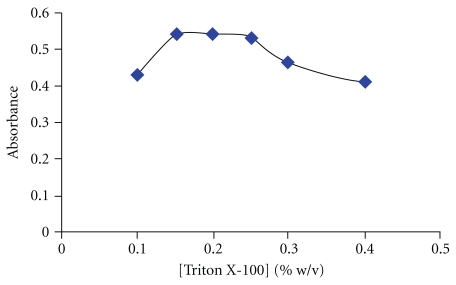
Effect of Triton X-100 concentration on the cloud point extraction. Conditions: 7.5 *μ*mol l^−1^ HDZ, 0.35 M HCl, equilibration time: 15 min, equilibration temperature: 40°C, and 50 ng mL^−1^ Se (IV).

The nonionic surfactant Triton X-100 was chosen because of its commercial availability of high purified homogeneous form, low toxicological properties, and cost. The cloud point temperature of Triton X-100 is 63.7°C [36]. To decrease the cloud point temperature of Triton X-100 to room temperature (desirable temperature in cloud point procedure), this solution was prepared by the diluent (solution of 0.5% (w/v) of phenol). The diluent decreases the cloud point of Triton X-100 to room temperature [37]. The variation of absorbance at *λ*_max_ of complex as a function of the concentration of Triton X-100 is shown in Figure 5. At lower concentrations of Triton X-100, the low extraction efficiency of the complex may be due to the inadequacy of the assemblies to entrap the hydrophobic complex quantitatively. The concentration of 0.2% (w/v) Triton X-100 was selected for further studies.

### 3.5. Equilibration Temperature and Time

**Figure 6:**
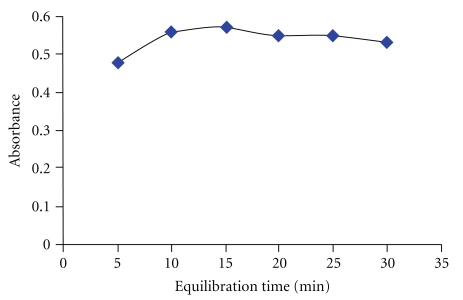
Effect of equilibration time on the complexation of Se (IV) with dithizone. Conditions: 0.2% (w/v) Triton X-100, 7.5 *μ*mol l^−1^ HDZ, 0.35 M HCl, equilibration temperature: 40°C and 50 ng mL^−1^ Se (IV).

**Figure 7:**
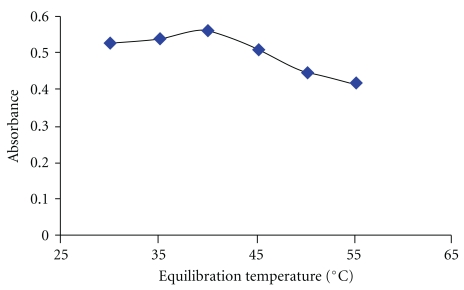
Effect of equilibration temperature on the corrected absorbance of the complex. Conditions: 0.2% (w/v) Triton X-100, 7.5 *μ*mol l^−1^ HDZ, 0.35 M HCl, equilibration time: 15 min, and 50 ng mL^−1^ Se (IV).

Optimal incubation time and equilibration temperature are necessary to complete the reaction, easy phase separation, and preconcentration as efficient as possible. Providing that the complexation reaction has been completed under certain conditions, corrected absorbance data show that, for equilibration times of 10 to 30 min, extraction efficiency is almost constant. The result of this investigation is shown in Figure 6. Hence, 15 min was chosen as optimal incubation time. On the other hand, it appears that the phase volume ratio of all nonionic surfactants decreases as the equilibration temperature increases. As can be seen in Figure 7, the corrected absorbance increases very gently up to 40°C and starts to decrease afterwards due to decrease in the stability of the dithizone and consequently the extraction efficiency. Hence, 40°C was chosen for further work.

The dependence of corrected absorbance upon centrifugation time was studied within the range of 5–30 min. It was found that a time of 15 min is adequate for separation of two phases.

### 3.6. Analytical Performance

Analytical characteristics for determination of selenium were studied under optimum conditions. Calibration graph was obtained by preconcentrating 50 mL of the sample containing 5, 15, 30, 40, 55, 70, 80, and 100 ng mL^−1^ of selenium (IV) under the optimal experimental conditions. Analytical parameters for the determination of the selenium-dithizonate complex are presented in Table 1. The experimental detection limit was found to be 4.4 ng mL^−1^. The relative standard deviation was 2.18% for six replicate measurements of standard selenium solution (50 ng mL^−1^). 

The effect of various cations and anions on the determination of 0.05 *μ*g mL^−1^ of Se(IV) was studied, and the results are summarized in Table 2. The tolerance limit was taken as the concentration of added ion causing less than 5% relative error. Cations such as Ag(I), Cu(II), Hg(II), and Fe(III) were interfered with the determination of Se(IV) ion. These cations can be removed using a cation exchange resin (sulfonic acid groups, mesh ranges: 50–100, 8 cm). For this purpose, 25 mL solution containing 2.5 *μ*g of Se(IV) and various amounts of desired cations was passed through 5 g cation exchange resin (8 cm wet resin) dropwise and collected in a 50 mL volumetric flask containing optimum amount of dithizone (7.5 *μ*mol l^−1^), HCl (0.35 M), and Triton X-100 (0.2% w/v). After washing the column with water (three times, with 15 mL), 0.5% (w/v) phenol was added and the solution was diluted to the mark with water and the amount of Se (IV) was determined using recommended procedure.

### 3.7. Application of the Proposed Method to Real Samples

To evaluate the analytical applicability, the method was applied to the determination of selenium in two multivitamin tablets (Centrum and Selen plus) and a selenium sulphide shampoo (for treatment of dandruff). The results for selenium sulphide shampoo, Centrum, and Selen plus multivitamins are given in Tables 3, 4, and 5, respectively. 

In order to evaluate the accuracy of procedure, selenium was determined in centrum tablet (containing 0.25 mg Se as sodium selenate) by ICP-OES as reference method. In both methods the results agreed well (Table 6). 

The limit of detection and relative standard deviation of the proposed method were compared with methods in the literature (Table 7). As observed from the results limit of detection by the proposed method was lower than other methods.

## 4. Conclusion

The simplicity, rapidity, and inexpensiveness of the proposed method in combination with the use of dithizone as a chromogenic reagent were utilized for selenium determination in cosmetic and pharmaceutical samples. Precision and recovery data clearly indicated the reproducibility and accuracy of the method. In this investigation, spectrophotometric determination of selenium was carried out in micellar medium without the need for an extraction step. Because of the overlapping of absorption maximum of complex at 424 nm with absorption maximum of dithizone at 434 nm, *A*_corr_ was applied. Cloud point extraction was used to improve the detection limit of this method.

## Figures and Tables

**Table 1:** Analytical parameters for determination of the selenium dithizonate complex.

Parameters	Se(HDZ)_4_ complex
*λ*_max_ (nm)	424
Beer's law range (ng mL^−1^)	5–100
Molar absorptivity (l mol^−1^ cm^−1^)	7.98 × 10^3^
LOD (ng mL^−1^)	4.4
LOQ (ng mL^−1^)	14.65
RSD (%)^a^	2.18
Linear regression equation (*y*)^b^	
Slop (*b*)	0.0095
Intercept (*a*)	0.0133
Correlation coefficient (*r*)	0.9981
*S*_*y*/*x*_	0.0146
*S*_*a*_	0.0098
*S*_*b*_	0.00017

^a^ *n* = 6.

^b^ *y* = *bx* + *a*, where *x* is the concentration in ng mL^−1^; *S*_*a*_: standard deviation of intercept.

*S*_*b*_: standard deviation of slop; *S*_*y*/*x*_: standard deviation of residuals.

**Table 2:** Tolerance limits of diverse ions on the determination of 50 ng mL^−1^ selenium at optimum conditions.

Ions	Tolerated ratio of foreign ion to Se(IV)
Na^+^, NO^−^_3_, NH_4_^+^, Mg^2+^, SO_4_^2−^, HPO_4_^2−^, H2PO_4_^−^, F^−^, Co^2+^	1000
Pb^2+^, Zn^2+^	800
Cu^2+^, Ag^+^, Hg^2+^, Fe^3+^	250^a^
Ni^2+^	200
SO_3_^−2^	5

^a^After removal with cation exchange resin.

**Table 3:** Determination of selenium in selenium sulphide shampoo.

Sample taken (mL)	Se (ng mL^−1^)		Recovery (%)	% RSD for triplicate measurements
	added	Found		
2	0	36.49	—	2.15
2	10	47.02	105.3	2.12
2	20	56.6	100.5	1.85
2	30	65.33	96.1	2.07

**Table 4:** Determination of selenium in Centrum tablet.

Sample taken (mL)	Se (ng mL^−1^)		Recovery (%)	% RSD for triplicate measurements
		added		
1	0	48.28	—	1.89
1	10	58.17	98.9	1.97
1	20	67.54	96.3	2.10
1	30	76.70	94.8	2.14

**Table 5:** Determination of selenium in Selen plus capsule.

Sample taken (mL)	Se (ng mL^−1^)		Recovery (%)	% RSD for triplicate measurements
		added		
1	0	37.54	—	1.88
1	10	47.33	97.9	2.08
1	20	56.70	95.8	1.93
1	30	66.18	95.4	2.12

**Table 6:** The results of selenium determination in Centrum tablet by the proposed and reference methods (*n* = 6).

Method	Label content of Se (mg)	Measured value (mg)
Proposed method	0.250	0.240
ICP-OES	0.250	0.245

**Table 7:** The results of limit of detection and relative standard deviation for several reported methods and proposed method.

Method	Limit of detection (ng mL^−1^)	Relative standard deviation (%)	Reference
INAA	17	5.0	[6]
Spectrophotometric	12	2.5	[28]
HG-AAS	10.6	1.93	[13]
ET-AAS	5	3.0	[11]
Proposed method	4.4	2.18	—
